# Dynamics and Epigenetics of the Epidermal Differentiation Complex

**DOI:** 10.3390/epigenomes8010009

**Published:** 2024-02-29

**Authors:** Wiesława Leśniak

**Affiliations:** Laboratory of Calcium Binding Proteins, Nencki Institute of Experimental Biology, Polish Academy of Sciences, 3 Pasteur St., 02-093 Warsaw, Poland; w.lesniak@nencki.edu.pl; Tel.: +48-22-5892-327

**Keywords:** epidermis, epidermal differentiation complex, epigenetics, DNA methylation, histone modifications

## Abstract

Epidermis is the outer skin layer built of specialized cells called keratinocytes. Keratinocytes undergo a unique differentiation process, also known as cornification, during which their gene expression pattern, morphology and other properties change remarkably to the effect that the terminally differentiated, cornified cells can form a physical barrier, which separates the underlying tissues from the environment. Many genes encoding proteins that are important for epidermal barrier formation are located in a gene cluster called epidermal differentiation complex (EDC). Recent data provided valuable information on the dynamics of the EDC locus and the network of interactions between EDC gene promoters, enhancers and other regions, during keratinocytes differentiation. These data, together with results concerning changes in epigenetic modifications, provide a valuable insight into the mode of regulation of EDC gene expression.

## 1. Introduction

Skin is the most external tissue that covers the body and provides a physical barrier separating and protecting it from the environment. The skin consists of three layers: hypodermis, dermis and epidermis. The latter, built of specialized cells called keratinocytes, forms the proper skin barrier that is highly impermeable and firm, but at the same time resilient. These unique properties of epidermis can be acquired through a process of epidermal differentiation during which keratinocytes synthesize specific lipids and proteins indispensible for barrier formation [1,2]. Like with any construction process, during which the building blocks have to be delivered on time and put to use in an ordered manner, the expression of genes encoding epidermal proteins is highly coordinated in time. How this coordination could be achieved was not known until an important clue was provided by the discovery that many epidermis-specific genes were grouped in clusters. For example, keratins, the main cytoskeletal elements of keratinocytes responsible for skin elasticity and resistance to mechanical stress, are encoded by genes organized in two gene clusters containing 26 and 28 genes, respectively, in the human genome [3]. Apart from keratins, many genes involved inepidermal cornification were found to form a cluster, known as the epidermal differentiation complex (EDC), on chromosome 1 in man and chromosome 3 in mouse [4]. Clustered gene organization may indicate that gene expression is regulated in the same manner throughout the whole cluster [5]. In this regard identification of the EDC gene cluster spurred a research effort aiming to determine how these genes were regulated and whether their expression was coordinated across the whole cluster, at the gene family level or individually. Since the process of epidermal differentiation is known to be under epigenetic control [6,7,8] much attention has been paid to epigenetic factors involved in regulation of EDC gene expression. Epigenetic factors shape the chromatin architecture to enable, or block, promoter-enhancer and other types of DNA interactions, which, in turn, control transcription. Since, thanks to recently developed methods, we gained a considerable insight into the spatial organization and dynamics of the locus it is interesting to track how these phenomena are interrelated in time and space and how they regulate EDC gene expression.

## 2. Epidermis

**Figure 1:**
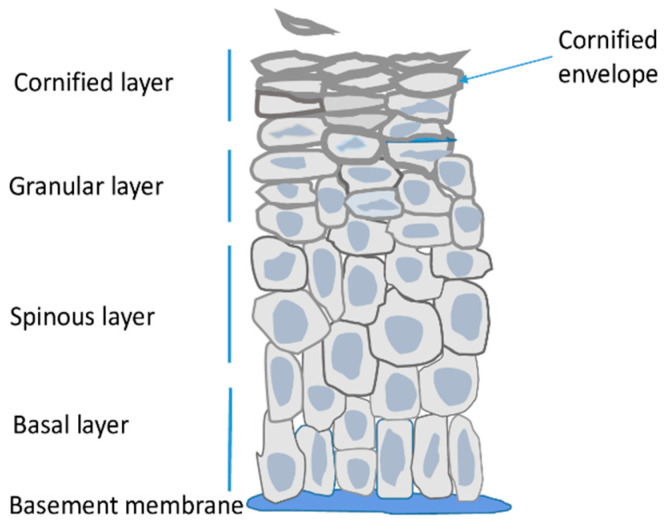
Schematic representation of the epidermis.

Epidermis is the outermost skin layer built of highly specialized cells called keratinocytes (Figure 1) [1]. It is separated from dermis by the basement membrane, that is, a layer of extracellular matrix rich in collagen IV and laminins, which are secreted by dermal fibroblasts and keratinocytes [9]. Keratinocytes originate from stem/progenitor cells present in the innermost layer of the epidermis termed the basal layer. Other resident cells i.e., melanocytes, Langerhans cells and Merkel cells constitute only a small fraction of epidermal cells, are of non-epithelial origin and perform other functions. Namely, they protect the skin against UV radiation, function in the immune system, and as tactile sensory cells, respectively [1,2].

The main role of epidermis is to serve as a tight but resilient barrier protecting the organism from water loss and external insults. In order to provide the barrier function keratinocytes of the basal layer undergo a series of morphological and metabolic changes during a process called terminal differentiation or cornification [1,2]. In the spinous layer keratinocytes cease to proliferate and start to synthesize large amounts of lipids, including ceramides and sterols, and of proteins, mainly keratins and other specialized epidermal proteins. In keratinocytes of the granular layer the synthesized proteins are stored in intracellular vesicles, so called keratohyalin granules, while lipids are present in lamellar bodies that later fuse with the plasma membrane and depose their lipid content outside the cell. The synthetized proteins become crosslinked through ε-(γ glutamyl) lysine (isopeptide) bonds by the action of transglutaminases. Expression of transglutaminases, as well as their activity, which is dependent on calcium concentration, are highest in the granular level of the epidermis [10]. The crosslinked proteins, together with lipids linked to them through esterification, form a thick, impermeable deposit, called the cornified envelope (CE), underneath the plasma membrane [2]. Formation of this sub-membranous structure defines the upper epidermal layer i.e., the cornified layer. Meanwhile, the cells become flattened, their nuclei and organelles deteriorate, the membranes disintegrate and the cornified envelopes of tightly connected cells, covered by extracellular lipids, form a continuous impermeable outer skin barrier (Figure 1). Those dead cells, also called corneocytes, gradually desquamate from the skin surface [2]. Epidermis is thus a constantly self-renewing tissue. In man, the life span of a keratinocyte is about 30–40 days during which the cell moves from the basal to the cornified layer and then desquamates [11].

During mouse embryogenesis epidermal stratification begins at day E14.5 and the functional epidermal barrier is formed at E18.5 [12]. Epidermal differentiation is a process synchronized both in time and space and its various stages can be traced by analyzing mRNA or protein expression pattern. Thus, for example, keratins 5 (K5) and 14 (K14) are mainly expressed in undifferentiated keratinocytes of the basal layer while K1 and K10 expression is characteristic for spinous and lower granular layer [2]. Subsequent differentiation stages are marked by high expression of structural proteins that are components of the cornified envelope such as involucrin or loricrin [2]. The overall transcriptional activity is highest in the basal and spinous layer keratinocytes and decreases significantly in the granular layer [13]. The drop in transcription rate is also reflected by an increasing number of heterochromatin clusters and lower level of active chromatin histone marks in differentiated keratinocytes [13,14].

## 3. Epidermal Differentiation Complex (EDC)

**Figure 2:**
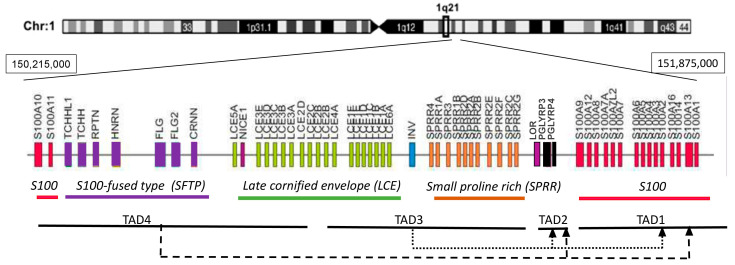
Schematic representation of the human epidermal differentiation complex. TAD—topologically associating domain. Dashed lines show contacts between TADs.

EDC is a gene cluster grouping structurally and functionally related genes involved in epidermal differentiation [15,16]. In man the cluster spans 1.6 Mb on chromosome 1 (1q21) while in mouse it extends over 3.1 Mb on chromosome 3 [4,17]. The locus is composed of four gene families and several individual genes, all of which are expressed in the epidermis [15,18]. The *S100* genes flank the locus on both sides (Figure 2). They encode small calcium-binding proteins with two EF-hand motifs that play various non-structural functions in the epidermis [19]. The most internal gene in the first *S100* subcluster, *S100A9*, is separated by two *Pglyrp* genes from the *Lor* gene encoding loricrin, and from *Sprr* genes encoding a family of small proline-rich (SPRR) proteins. Loricrin and SPRR proteins are the principal components of the cornified envelope constituting 70–85% of its mass [20,21]. Their domain structure, which is common for all EDC proteins with the exception of S100 proteins, includes a central part consisting of repetitive sequences and the N- and C-terminal domains usually rich in lysine and glutamine residues. In SPRR proteins, the repetitive sequences contain many proline residues [22] while in loricrin they abound with Gln, Cys, Ser [2]. All these proteins are transglutaminase substrates, with SPRR proteins probably acting as cross-bridging molecules for loricrin. The late cornified envelope (*Lce*) genes, divided into several subgroups, code for proteins that, like SPRR proteins, probably serve as cross-bridging proteins for CE components [23,24]. The involucrin (*Ivl*) gene encodes a rod-like protein, rich in glutamine and glutamic acid, that may have a role in the initial phase of CE assembly since it is expressed relatively early during differentiation [25]. S100-fused type (*Sftp*) genes encode 8 proteins, among them filaggrin, cornerin or trichohyalin. Like S100 proteins, they possess two EF-hand structures but also repeat units similarly to other EDC encoded proteins [15]. SFTP proteins are present in the cytoplasm and probably bundle the keratin filaments and/or cross-bridge them to CE.

Based on comparative genomics it can be speculated that the EDC genes evolved in amniotes, in strict association with land dwelling, from two founding genes, encoding the ancestral S100 and PGLYRP proteins, through fusion, insertion, inversion and multiple duplication and deletion events [26]. With few exceptions the EDC genes preserved a similar exon/intron structure and are highly conserved between species. Namely, they contain either 2 exons (e.g., *SPRR*s, most *LCE* genes, *Lor*) or 3 exons, of which the first is not translated (e.g., *S100*) [20,27]. Except for the *S100* genes, some of which are expressed in undifferentiated cells in the basal layer [19,28,29], the majority of EDC genes are expressed at later stages of epidermal differentiation. Accordingly, although the overall transcription rate drops during keratinocyte differentiation, EDC gene expression is highest in differentiated cells [29].

## 4. Chromatin Dynamics within the EDC Locus during Embryogenesis and Differentiation

It is generally accepted that location of a given gene in the interphase nucleus is non-random but tightly associated with its transcriptional activity, with actively transcribed genes tending to locate closer to the center of the nucleus and protrude from the chromosome territory [30,31]. Since EDC represents tissue-specific genes with largely time-coordinated expression it was interesting to establish whether transcriptional activation of EDC genes is mirrored by changes in locus position. Studies employing 2D and 3D fluorescence in situ hybridization (FISH) analysis established that the EDC bearing chromosomes, chromosome 1 in man and chromosome 3 in mouse, were located at the nuclear periphery and did not change position during embryonic development or epidermal differentiation [13,32,33]. However, the EDC locus itself appeared to behave in a much more dynamic way. Namely, in differentiating primary human keratinocytes, its central part (covering *Ivl*, *Sprr* and *Lor*) was often observed looping out of the chromosomal territory and facing the nuclear interior [32]. This external localization of EDC could also be observed, but to a lesser extent, in undifferentiated keratinocytes of the basal layer. Interestingly, the EDC segment containing *S100A1-S100A9* was slightly more exposed in basal than differentiated keratinocytes in accordance with the expression pattern. No looping out of the EDC region was detected in lymphoblastoid cells, in which this locus was not actively transcribed [32]. Similar dynamics of the EDC locus could be observed during mouse embryonic development. Namely, before epidermal stratification (E11.5), the locus was visible at the peripheral part of chromosome 3 territory, close to the nuclear membrane [33]. At E16,5, during stratification, the localization shifted to the internal part of the territory facing the nuclear center, in close vicinity to nuclear speckles rich in RNA processing factors. No such shift was observed in dermal cells [33]. Those changes in EDC dynamics were dependent on the p63 transcription factor, a major regulator of epidermal development [34], acting via its direct target genes encoding chromatin remodelers such as Satb1 [35] or Brg1 [33].

Another level of EDC dynamics, involving intra-chromatin interactions within and outside the gene cluster, was investigated using 3C or 5C (Chromatin Conformation Copy or Chromatin Conformation Capture Carbon Copy) technology, designed to trace the spatial organization of chromatin and physical contacts between various chromatin regions [36]. This technology makes it possible to map topologically associating domains (TADs), that is chromatin domains within which spatial interactions between DNA sequences are more frequent than with sequences outside the domain. TADs usually contain both genes and their enhancers, and are believed to represent functional units of chromatin. DNA contacts within TADs are facilitated by DNA strand loop formation and other spatial reorganizations that reduce the distances separating particular DNA sequences in linear DNA [37,38].

The use 3C and 5C technology led to the identification of four TADs in EDC (Figure 2) [39]. A gene rich TAD1 spans the *S1001-S100A9* genes and some genes beyond the EDC boundary. A gene poor TAD2 encompasses the gene-poor distance between the *S100* genes and the *Lor* gene. TAD3 contains the *Sprr* genes, the *Ivl* gene, and the major part of the *Lce* gene family, and TAD4 encompasses the rest of the *Lce* genes, *Sftp* genes and the *S100A10-S100A11* genes [39]. Interestingly, in addition to multiple spatial interactions within each TAD, the analysis traced many spatial contacts between various TADs in EDC and beyond. In particular the gene-rich TAD3 and TAD4 made frequent contacts with TAD1 and the gene-poor TAD2 [Figure 2]. The latter, on the other hand, showed intense interactions with gene-poor TAD5, outside of the EDC boundary. The enhancers were specifically enriched in TAD1, which harbored seven of the eleven enhancers identified within EDC [39]. Most of the enhancers were engaged in long distance contacts with gene promoters located also in other TADs. For example, in differentiating keratinocytes, the 923 enhancer located near the *Ivl* gene in TAD3 made spatial contact with *Ivl*, several *Lce* and *Sprr* genes but also with *S100A6* located in TAD1 [40]. Conversely, many gene promoters interacted with several enhancers including enhancers located outside EDC [39]. Further studies established that the looping pattern and the frequency of enhancer-promoter contacts differed between undifferentiated keratinocytes of the basal layer and differentiating cells in suprabasal epidermal layers [29,40]. While some of the spatial contacts were stable over time many other were altered and the looping intensity became higher in suprabasal keratinocytes. The increase in DNA dynamics as well as a higher number of open chromatin compartments observed in EDC of differentiated cells matched the higher transcriptional activity of this locus [29]. Altogether, the boundaries between TADs and spatial contacts within and between TADs were much more conspicuous in keratinocytes than in thymocytes suggesting that the chromatin structure of the EDC region differed considerably between these two cell types [39].

## 5. Epigenetic Factors Involved in EDC Gene Expression Regulation

Results described above show the EDC gene cluster as a highly dynamic genomic region that becomes exposed to the nucleoplasm and engages in intense internal and external interactions when keratinocytes start to differentiate. Such flexibility of chromatin requires an appropriate epigenetic landscape that can be introduced by epigenetic modifications. The latter include DNA methylation and various modifications (e.g., acetylation, methylation) of lysine, and also arginine, residues in the N-termini of histone molecules [41]. In general, cytosine methylation in DNA favors chromatin condensation while the effect of histone modifications depends on the type of modification and the modified residue. These epigenetic marks can attract chromatin remodeling complexes that alter the chromatin architecture and change its accessibility to transcription factors. The currently available data offer some insight into how the pattern of epigenetic modifications changes during keratinocyte differentiation to enable both active transcription of EDC genes and an adequate transcriptional control.

### 5.1. DNA Methylation

Results obtained on keratinocytes isolated from neonatal skin, which have a high renewal potential and are enriched in progenitor cells, indicated that DNA methylation was indispensable for epidermal homeostasis, particularly for maintaining the balance between stem cell renewal, proliferation and subsequent differentiation. Notably, knock-down of the maintenance DNA methyltransferase (DNMT1) that methylates the newly synthetized DNA strand following replication, impaired the function of progenitor cells as reflected by a fewer number of proliferating cells, thinner epidermal layer and premature differentiation [42]. The impairment of progenitor cell function was also evident in K14-Cre transgenic mice with DNMT1 knockout. The K14-Cre mouse model, expressing the Cre recombinase under control of the *K14* gene promoter allows for targeting the expression of genes in the basal layer of mouse epidermis and is extremely useful in studying the impact of various genetic factors on epidermal differentiation [43]. In this regard, epidermis of K14-DNMT1-KO mice showed abnormal stem cell activity in the interfollicular epidermis and hair follicles and premature differentiation exemplified, among others, by an early expression of an EDC gene encoding involucrin [44].

Aside from the impairment of progenitor cell function DNMT1 knockdown in cultured keratinocyte led to altered gene expression [42]. Gene ontology (GO) analysis showed that DNMT1 deficiency resulted in repressed expression of proliferation-associated genes and enhanced expression of differentiation genes. Among differentiation genes induced upon DNMT1 knockdown were several EDC genes: *Ivl*, *Lce3D*, four *S100* genes (*S100A4*, *S100A7*, *S100A8*, *S100A9*) and some *SPRR* genes, suggesting that these genes may be regulated by DNA methylation. On the other hand, depletion of GADD45, a protein involved in the repair-based DNA demethylation, led to reduced expression of differentiation genes, among them *Ivl*, *Lce3D*, *Sprr3* or *S100A8*, while overexpression had a contrary effect [42]. In correspondence with the above data, knockout of DNA demethylase, TET2, in the basal epidermal layer, resulted in suppression of differentiation genes, including a number of EDC genes (*Flg*, *Rptn*, *Lce1G*, *Sprr1B*, *Sprr2a1* and *Sprr2a3*) [45]. However, MeDIP (methylated DNA immunoprecipitation) analysis of methylation peaks showed that among the EDC genes only *LCE3D* had altered methylation status, that is, became demethylated in differentiated keratinocytes [42]. This could indicate that altered expression of enzymes involved in DNA methylation/demethylation affected the expression of EDC genes indirectly, without causing any changes in their methylation status. Indeed, results obtained using primary keratinocytes derived from adult human donors showed only a limited impact of DNA methylation on the expression of differentiation genes. In particular none of the EDC genes examined (*S100A6*, *S100A7*, *S100A8*, *S100A13*, *Inv*, *Lor*, *Nice1*) revealed an altered promoter methylation status during in vitro differentiation in spite of extensive changes in expression [46]. Likewise, no major changes in DNA methylation were detected in selected EDC regions by targeted NGS [47]. Lack of changes in the methylation status in EDC and, in fact, in the whole keratinocyte genome during differentiation, was confirmed using a methylation array covering 850,000 genomic loci [48]. Altogether, these results indicate that changes in the pattern of DNA methylation, priming the epigenetic landscape for subsequent changes in EDC gene expression, probably occur very early in epidermal development and precede epidermal differentiation.

### 5.2. Histone Modifications

Histones are structural DNA-binding proteins that confer a primary structure to chromatin by organizing it into nucleosomes. Additionally, covalent posttranslational modifications (PTMs) of their basic N-termini can attract architectural proteins and chromatin remodeling complexes which, in turn, determine the higher order structure of chromatin and can render it more or less accessible for transcription [41,49]. While the mechanism of chromatin structure remodeling is common for all such complexes the direction of changes in chromatin accessibility depends on the type of histone PTMs. Acetylation of basic lysine residues by histone acetyltransferases (HAT) is associated with transcriptionally active chromatin while removal of the acetyl group by deacetylases (HDAC) correlates with transcription inhibition [50]. Contrary to that, the effect of lysine or arginine methylation, introduced by histone methyltransferases (HMT) depends on the position of the modified residue. Di- or trimethylation of H3K9, H3K27 or H4K20 have a repressive effect on transcription while trimethylation of H3K4, H3K56 or H3K results in higher gene expression. Removal of the methyl group by histone demethylases (HDMs) reverses the effect of methylation. All histone modifications are reversible therefore they can shape the chromatin structure in a very dynamic way to meet the needs of various cellular processes.

Studies performed on postnatal or embryonic mouse epidermis undergoing stratification (E14.5–E18.5) or on adult primary keratinocytes subjected to differentiation in vitro revealed important differences in the pattern of histone modifications between the basal layer, containing stem and undifferentiated cells, and suprabasal epidermis comprising the upper epidermal layers (Figure 1). More precisely, immunohistochemical staining showed more abundant presence of H3K4me3 and H3K27ac, the activating histone marks, in the basal layer [14,51]. A more intense signal for histone H4 acetylated at lysines 12 and 16 (H4K12ac, H4K16ac) was also detected in the basal compared with the suprabasal layer [51] although low overall H4 acetylation in the basal layer was observed in another study [52]. On the other hand the suprabasal epidermis was enriched in repressive histone marks i.e., H3K27me3 and H4K20me3. Another repressive histone mark, H3K9me3, was present in both studied epidermal compartments either at equal levels or was more abundant in the basal layer [51,52]. Thus, the spatial distribution of histone modifications, with the basal layer enriched in activating histone modifications and the repressive histone marks prevailing in the suprabasal layers, seems to agree with the overall transcription rate, which is higher in undifferentiated basal layer keratinocytes [29].

The type and distribution of histone modifications on EDC genes have not been studied systematically but there are nonetheless some interesting data concerning this aspect of their regulation. For example, chromatin immunoprecipitation (ChIP) results showed the presence of an inhibitory histone mark, H3K27me3, on the promoters of *Ivl*, *Flg*, *S100A8* and *Lce* in basal layer keratinocytes, and its subsequent loss upon differentiation [53,54]. Interestingly, H3K27me3 was shown to be practically absent from the *Sprr* gene promoters. These observations were corroborated by studies on animals with conditional depletion of histone modifying enzymes in the basal epidermal layer. Knockout of Ezh2, a H3K27 methyltransferase, resulted in the loss of H3K27me3 at promoters of many differentiation genes and led to increased expression of *Lor*, *Flg*, *Ivl* and *Lce* genes and to precocious epidermal differentiation [53]. As could be expected, the *Sprr* genes were not upregulated by Ezh2 knockout indicating another mechanism of their activation. A similar phenomenon i.e., premature differentiation and increased expression of some EDC genes (*Lor*, *Flg*, *Lce1a*, *Lce1c*, *Lce1d*, *Lce1l*), was observed in neonatal mouse epidermis with a knockout of Jarid2, which, like Ezh2, is a component of the repressive PRC2 complex [55]. Furthermore, a similar course of events, that is premature differentiation, was observed in primary keratinocytes overexpressing H3K27 demethylase, Jmjd3 [54]. This coincided with higher expression of *S100A8*, *Flg* and *Ivl*. On the other hand, Jmjd3 silencing inhibited expression of those genes and retarded differentiation. Knockout of another histone methyltransferase, SUV39H1, which imposes a repressive chromatin mark by methylating H3K9, also induced some (*S100A8*, *Lce1A*, *Lce1B*, *Lce1C*) but remained without effect on other (*Flg*, *Lor*, *Ivl*) EDC genes in undifferentiated HaCaT keratinocytes [56]. The induction was not observed in differentiated cells, in which those genes were already actively transcribed.

Definitely less is known about the role of histone acetylation in EDC gene expression. Increased expression of *Sprr1*, *Sprr2* and *Ivl* was observed in primary keratinocytes treated with sodium butyrate, a histone deacetylase inhibitor, suggesting that these genes could be activated by histone acetylation [28]. However, conditional depletion of histone deacetylases, HDAC1 and HDAC2, in mouse embryonal epidermis appeared to inhibit the expression of another EDC gene, *Lor*, and disturbed epidermal stratification [57]. Another study showed that diminished expression of a histone acetyltransferase, KAT2B, led to inhibition of *Ivl*, *Flg* and *Lce1A* and of other differentiation genes especially in confluent i.e., differentiated keratinocytes, in which the enzyme is expressed at a higher level [58]. On the other hand, silencing of KAT2A, induced differentiation of subconfluent (undifferentiated) keratinocytes and promoted expression of *Ivl*, *Flg* and *Lce*1A, and of other differentiation genes [58]. This effect was much less pronounced when cells differentiated. KAT2A silencing reduced the overall level of acetylated histone H3 (H3ac) and, specifically, the level of H3K9ac. Since changes in the H3ac histone mark on the promoters of differentiation genes has not been studied it is difficult to resolve if the effect of KAT2A silencing on their expression was direct or non-direct. The latter seems more probable based on the recognized role of KAT2A in control of cell renewal and differentiation [58].

Results presented above show the importance of the integrity and stability of histone modifications at each stage of epidermal differentiation. Expression of many, but probably not all, EDC genes is suppressed in undifferentiated cells by the presence of repressive histone marks (H3K27me3, H3K9me3) on their promoters. Upon differentiation this repression is released probably due to decreased histone methyltransferase expression [53] and/or the action of histone acetyltransferases.

## 6. Conclusions

The discovery that many epidermal differentiation genes formed a gene cluster raised important questions regarding (1) regulation of their expression and (2) the extent of coordination of their expression i.e., whether it was coordinated throughout the whole EDC cluster, individually or, possibly, at the gene family level. As to expression regulation, subsequent research effort led to identification of numerous transcription factors involved in EDC gene transcription and regulation [15]. Also, the role of epigenetic factors in transcriptional activation of the locus has been examined. Based on the available data it can be speculated that DNA methylation/demethylation does not play an active role in control of EDC gene expression during differentiation. Conversely, histone modifications and, in particular, the release of inhibitory histone marks H3K27me3 and H3K9me3, may trigger the subsequent changes in EDC chromatin that facilitate active transcription and also, via spatial interactions, ensure its proper control.

As regards coordination of EDC gene expression, results of initial studies by Elder and Zhao suggested lack of global gene expression regulation or coordination across the whole EDC [28]. It was however postulated that spatial proximity of some genes could favor their coordinate regulation. Data available so far seem to support this conclusion. In particular, it is evident that the inhibitory histone marks, H3K27me3 or H3K9me3, do not associate with all EDC gene promoters in undifferentiated keratinocytes and that their removal activates transcription of only certain (e.g., *Lce*) but not all genes (e.g., *Sprrs*) [53]. At the same time the results seem to support the notion that there is some extent of expression coordination at the level of gene families e.g., *Lce* [53,55]. The multiplicity and diversity of spatial interactions within EDC, showing that neighboring genes can receive inputs from different enhancers and that a given enhancer interacts with only certain members of a gene family [39,40], also favor the conclusion that there is much individuality in EDC gene expression regulation. Thus, it seems that a combination of diverse regulatory events rather than a uniform pattern of regulation controls EDC gene expression.

## Abbreviations

CE, cornified envelope; EDC, epidermal differentiation complex; FISH, fluorescence in situ hybridization; Ivl, involucrin; K1, keratin 1; K10, keratin 10; K14, keratin 14; LCE, late cornified envelope proteins; Lor, loricrin; SFTP, S100-fused type proteins; SPRR, small proline rich proteins; TAD, topologically associating domain.

## References

[1] Simpson C.L., Patel D.M., Green K.J. (2011). Deconstructing the skin: Cytoarchitectural determinants of epidermal morphogenesis. Nat. Rev. Mol. Cell Biol..

[2] Candi E., Schmidt R., Melino G. (2005). The cornified envelope: A model of cell death in the skin. Nat. Rev. Mol. Cell Biol..

[3] Schweizer J., Bowden P.E., Coulombe P.A., Langbein L., Lane E.B., Magin T.M., Maltais L., Omary M.B., Parry D.A., Rogers M.A. (2006). New consensus nomenclature for mammalian keratins. J. Cell Biol..

[4] Ridinger K., Ilg E.C., Niggli F.K., Heizmann C.W., Schafer B.W. (1998). Clustered organization of S100 genes in human and mouse. Biochim. Biophys. Acta.

[5] Hurst L.D., Pal C., Lercher M.J. (2004). The evolutionary dynamics of eukaryotic gene order. Nat. Rev. Genet..

[6] Perdigoto C.N., Valdes V.J., Bardot E.S., Ezhkova E. (2014). Epigenetic regulation of epidermal differentiation. Cold Spring Harb. Perspect. Med..

[7] Moltrasio C., Romagnuolo M., Marzano A.V. (2022). Epigenetic Mechanisms of Epidermal Differentiation. Int. J. Mol. Sci..

[8] Leśniak W. (2021). Epigenetic regulation of epidermal differentiation. Epigenomes.

[9] Aumailley M. (2021). Laminins and interaction partners in the architecture of the basement membrane at the dermal-epidermal junction. Exp. Dermatol..

[10] Eckert R.L., Sturniolo M.T., Broome A.M., Ruse M., Rorke E.A. (2005). Transglutaminases in epidermis. Prog. Exp. Tumor Res..

[11] Weinstein G.D., McCullough J.L., Ross P. (1984). Cell proliferation in normal epidermis. J. Investig. Dermatol..

[12] Bazzi H., Fantauzzo K.A., Richardson G.D., Jahoda C.A., Christiano A.M. (2007). Transcriptional profiling of developing mouse epidermis reveals novel patterns of coordinated gene expression. Dev. Dyn..

[13] Gdula M.R., Poterlowicz K., Mardaryev A.N., Sharov A.A., Peng Y., Fessing M.Y., Botchkarev V.A. (2013). Remodeling of three-dimensional organization of the nucleus during terminal keratinocyte differentiation in the epidermis. J. Investig. Dermatol..

[14] Dube C.T., Jahan F.R.S., Lim C.Y. (2022). Key changes in chromatin mark mammalian epidermal differentiation and ageing. Epigenetics.

[15] Kypriotou M., Huber M., Hohl D. (2012). The human epidermal differentiation complex: Cornified envelope precursors, S100 proteins and the ‘fused genes’ family. Exp. Dermatol..

[16] Oh I.Y., de Guzman Strong C. (2017). The Molecular Revolution in Cutaneous Biology: EDC and Locus Control. J. Investig. Dermatol..

[17] Mischke D., Korge B.P., Marenholz I., Volz A., Ziegler A. (1996). Genes encoding structural proteins of epidermal cornification and S100 calcium-binding proteins form a gene complex (“epidermal differentiation complex”) on human chromosome 1q21. J. Investig. Dermatol..

[18] Henry J., Toulza E., Hsu C.Y., Pellerin L., Balica S., Mazereeuw-Hautier J., Paul C., Serre G., Jonca N., Simon M. (2012). Update on the epidermal differentiation complex. Front. Biosci. (Landmark Ed.).

[19] Leśniak W., Graczyk-Jarzynka A. (2015). The S100 proteins in epidermis: Topology and function. Biochim. Biophys. Acta.

[20] Cabral A., Voskamp P., Cleton-Jansen A.M., South A., Nizetic D., Backendorf C. (2001). Structural organization and regulation of the small proline-rich family of cornified envelope precursors suggest a role in adaptive barrier function. J. Biol. Chem..

[21] Steinert P.M., Marekov L.N. (1995). The proteins elafin, filaggrin, keratin intermediate filaments, loricrin, and small proline-rich proteins 1 and 2 are isodipeptide cross-linked components of the human epidermal cornified cell envelope. J. Biol. Chem..

[22] Carregaro F., Stefanini A.C.B., Henrique T., Tajara E.H. (2013). Study of small proline-rich proteins (SPRRs) in health and disease: A review of the literature. Arch. Dermatol. Res..

[23] Marshall D., Hardman M.J., Nield K.M., Byrne C. (2001). Differentially expressed late constituents of the epidermal cornified envelope. Proc. Natl. Acad. Sci. USA.

[24] Jackson B., Tilli C.M., Hardman M.J., Avilion A.A., MacLeod M.C., Ashcroft G.S., Byrne C. (2005). Late cornified envelope family in differentiating epithelia--response to calcium and ultraviolet irradiation. J. Investig. Dermatol..

[25] Eckert R.L., Yaffe M.B., Crish J.F., Murthy S., Rorke E.A., Welter J.F. (1993). Involucrin-structure and role in envelope assembly. J. Investig. Dermatol..

[26] Strasser B., Mlitz V., Hermann M., Rice R.H., Eigenheer R.A., Alibardi L., Tschachler E., Eckhart L. (2014). Evolutionary origin and diversification of epidermal barrier proteins in amniotes. Mol. Biol. Evol..

[27] Marenholz I., Volz A., Ziegler A., Davies A., Ragoussis I., Korge B.P., Mischke D. (1996). Genetic analysis of the epidermal differentiation complex (EDC) on human chromosome 1q21: Chromosomal orientation, new markers, and a 6-Mb YAC contig. Genomics.

[28] Elder J.T., Zhao X. (2002). Evidence for local control of gene expression in the epidermal differentiation complex. Exp. Dermatol..

[29] Nayak S., Jiang K., Hope E., Cross M., Overmiller A., Naz F., Worrell S., Bajpai D., Hasneen K., Brooks S.R. (2023). Chromatin Landscape Governing Murine Epidermal Differentiation. J. Investig. Dermatol..

[30] Cremer T., Cremer M. (2010). Chromosome territories. Cold Spring Harb. Perspect. Biol..

[31] Bickmore W.A. (2013). The spatial organization of the human genome. Annu. Rev. Genom. Hum. Genet..

[32] Williams R.R., Broad S., Sheer D., Ragoussis J. (2002). Subchromosomal positioning of the epidermal differentiation complex (EDC) in keratinocyte and lymphoblast interphase nuclei. Exp. Cell Res..

[33] Mardaryev A.N., Gdula M.R., Yarker J.L., Emelianov V.U., Poterlowicz K., Sharov A.A., Sharova T.Y., Scarpa J.A., Joffe B., Solovei I. (2014). p63 and Brg1 control developmentally regulated higher-order chromatin remodelling at the epidermal differentiation complex locus in epidermal progenitor cells. Development.

[34] Truong A.B., Khavari P.A. (2007). Control of keratinocyte proliferation and differentiation by p63. Cell Cycle.

[35] Fessing M.Y., Mardaryev A.N., Gdula M.R., Sharov A.A., Sharova T.Y., Rapisarda V., Gordon K.B., Smorodchenko A.D., Poterlowicz K., Ferone G. (2011). p63 regulates Satb1 to control tissue-specific chromatin remodeling during development of the epidermis. J. Cell. Biol..

[36] Dostie J., Richmond T.A., Arnaout R.A., Selzer R.R., Lee W.L., Honan T.A., Rubio E.D., Krumm A., Lamb J., Nusbaum C. (2006). Chromosome Conformation Capture Carbon Copy (5C): A massively parallel solution for mapping interactions between genomic elements. Genome Res..

[37] Dixon J.R., Gorkin D.U., Ren B. (2016). Chromatin Domains: The unit of chromosome organization. Mol. Cell..

[38] Long H.S., Greenaway S., Powell G., Mallon A.M., Lindgren C.M., Simon M.M. (2022). Making sense of the linear genome, gene function and TADs. Epigenetics Chromatin.

[39] Poterlowicz K., Yarker J.L., Malashchuk I., Lajoie B.R., Mardaryev A.N., Gdula M.R., Sharov A.A., Kohwi-Shigematsu T., Botchkarev V.A., Fessing M.Y. (2017). 5C analysis of the Epidermal Differentiation Complex locus reveals distinct chromatin interaction networks between gene-rich and gene-poor TADs in skin epithelial cells. PLoS Genet..

[40] Oh I.Y., Albea D.M., Goodwin Z.A., Quiggle A.M., Baker B.P., Guggisberg A.M., Geahlen J.H., Kroner G.M., de Guzman Strong C. (2014). Regulation of the dynamic chromatin architecture of the epidermal differentiation complex is mediated by a c-Jun/AP-1-modulated enhancer. J. Investig. Dermatol..

[41] Swygert S.G., Peterson C.L. (2014). Chromatin dynamics: Interplay between remodeling enzymes and histone modifications. Biochim. Biophys. Acta.

[42] Sen G.L., Reuter J.A., Webster D.E., Zhu L., Khavari P.A. (2010). DNMT1 maintains progenitor function in self-renewing somatic tissue. Nature.

[43] Vasioukhin V., Degenstein L., Wise B., Fuchs E. (1999). The magical touch: Genome targeting in epidermal stem cells induced by tamoxifen application to mouse skin. Proc. Natl. Acad. Sci. USA.

[44] Li J., Jiang T.X., Hughes M.W., Wu P., Yu J., Widelitz R.B., Fan G., Chuong C.M. (2012). Progressive alopecia reveals decreasing stem cell activation probability during aging of mice with epidermal deletion of DNA methyltransferase 1. J. Investig. Dermatol..

[45] Boudra R., Woappi Y., Wang D., Xu S., Wells M., Schmults C.D., Lian C.G., Ramsey M.R. (2022). Regulation of 5-Hydroxymethylcytosine by TET2 contributes to squamous cell carcinoma tumorigenesis. J. Investig. Dermatol..

[46] Sobiak B., Graczyk-Jarzynka A., Leśniak W. (2016). Comparison of DNA Methylation and Expression Pattern of S100 and Other Epidermal Differentiation Complex Genes in Differentiating Keratinocytes. J. Cell. Biochem..

[47] Sobiak B., Leśniak W. (2019). The Effect of Single CpG Demethylation on the Pattern of DNA-Protein Binding. Int. J. Mol. Sci..

[48] Smits J.P.H., Dirks R.A.M., Qu J., Oortveld M.A.W., Brinkman A.B., Zeeuwen P.L.J.M., Schalkwijk J., Zhou H., Marks H., van den Bogaard E.H. (2021). Terminal keratinocyte differentiation in vitro is associated with a stable DNA methylome. Exp. Dermatol..

[49] Luger K., Dechassa M.L., Tremethick D.J. (2012). New insights into nucleosome and chromatin structure: An ordered state or a disordered affair?. Nat. Rev. Mol. Cell Biol..

[50] Marmorstein R., Zhou M.M. (2014). Writers and readers of histone acetylation: Structure, mechanism, and inhibition. Cold Spring Harb. Perspect. Biol..

[51] Shue Y.T., Lee K.T., Walters B.W., Ong H.B., Silvaraju S., Lam W.J., Lim C.Y. (2020). Dynamic shifts in chromatin states differentially mark the proliferative basal cells and terminally differentiated cells of the developing epidermis. Epigenetics.

[52] Frye M., Fisher A.G., Watt F.M. (2007). Epidermal stem cells are defined by global histone modifications that are altered by Myc-induced differentiation. PLoS ONE.

[53] Ezhkova E., Pasolli H.A., Parker J.S., Stokes N., Su I.H., Hannon G., Tarakhovsky A., Fuchs E. (2009). Ezh2 orchestrates gene expression for the stepwise differentiation of tissue-specific stem cells. Cell.

[54] Sen G.L., Webster D.E., Barragan D.I., Chang H.Y., Khavari P.A. (2008). Control of differentiation in a self-renewing mammalian tissue by the histone demethylase JMJD3. Genes Dev..

[55] Mejetta S., Morey L., Pascual G., Kuebler B., Mysliwiec M.R., Lee Y., Shiekhattar R., Di Croce L., Benitah S.A. (2011). Jarid2 regulates mouse epidermal stem cell activation and differentiation. EMBO J..

[56] Sobiak B., Leśniak W. (2020). Effect of SUV39H1 Histone Methyltransferase Knockout on Expression of Differentiation-Associated Genes in HaCaT Keratinocytes. Cells.

[57] LeBoeuf M., Terrell A., Trivedi S., Sinha S., Epstein J.A., Olson E.N., Morrisey E.E., Millar S.E. (2010). Hdac1 and Hdac2 act redundantly to control p63 and p53 functions in epidermal progenitor cells. Dev. Cell.

[58] Walters B.W., Tan T.J., Tan C.T., Dube C.T., Lee K.T., Koh J., Ong Y.H.B., Tan V.X.H., Jahan F.R.S., Lim X.N. (2023). Divergent functions of histone acetyltransferases KAT2A and KAT2B in keratinocyte self-renewal and differentiation. J. Cell. Sci..

